# NK Cells Contribute to the Immune Risk Profile in Kidney Transplant Candidates

**DOI:** 10.3389/fimmu.2019.01890

**Published:** 2019-08-23

**Authors:** David DeWolfe, Malika Aid, Kevin McGann, Joshua Ghofrani, Emma Geiger, Catherine Helzer, Shaily Malik, Steve Kleiboeker, Stephanie Jost, Chen Sabrina Tan

**Affiliations:** ^1^Transplant Institute, Beth Israel Deaconess Medical Center, Harvard Medical School, Boston, MA, United States; ^2^Center for Virology and Vaccines Research, Beth Israel Deaconess Medical Center, Harvard Medical School, Boston, MA, United States; ^3^Viracor-Eurofins, Lee's Summit, MO, United States; ^4^Division of Infectious Diseases, Beth Israel Deaconess Medical Center, Harvard Medical School, Boston, MA, United States

**Keywords:** NK cells, immune risk profile, BK virus, kidney transplant, immune senescence, end stage renal disease, dialysis

## Abstract

**Background:** A previously proposed immune risk profile (IRP), based on T cell phenotype and CMV serotype, is associated with mortality in the elderly and increased infections post-kidney transplant. To evaluate if NK cells contribute to the IRP and if the IRP can be predicted by a clinical T cell functional assays, we conducted a cross sectional study in renal transplant candidates to determine the incidence of IRP and its association with specific NK cell characteristics and ImmuKnow® value.

**Material and Methods:** Sixty five subjects were enrolled in 5 cohorts designated by age and dialysis status. We determined T and NK cell phenotypes by flow cytometry and analyzed multiple factors contributing to IRP.

**Results:** We identified 14 IRP+ [CMV seropositivity and CD4/CD8 ratio < 1 or being in the highest quintile of CD8+ senescent (28CD–/CD57+) T cells] individuals equally divided amongst the cohorts. Multivariable linear regression revealed a distinct IRP+ group. Age and dialysis status did not predict immune senescence in kidney transplant candidates. NK cell features alone could discriminate IRP– and IRP+ patients, suggesting that NK cells significantly contribute to the overall immune status in kidney transplant candidates and that a combined T and NK cell phenotyping can provide a more detailed IRP definition. ImmuKnow® value was negatively correlated to age and significantly lower in IRP+ patients and predicts IRP when used alone or in combination with NK cell features.

**Conclusion:** NK cells contribute to overall immune senescence in kidney transplant candidates.

## Introduction

The number of elderly end stage renal disease (ESRD) patients in the United States continues to grow (1), as does the number of elderly patients listed for kidney transplant (2, 3). Several studies have shown impaired T-cell function in both ESRD (4–6) and elderly individuals (7, 8) and that these conditions compound one another (9, 10). Elderly recipients appear to have a lower risk of allograft rejection; however, they are at an increased risk for infectious complications after transplant. Lowering of immune suppressions to treat infections can often precipitate rejection episodes with detrimental outcomes (11–13), highlighting the fine line that must be walked in immune suppressing such patients.

Changes in T-cell phenotype and function known as T-cell senescence have been associated with mortality in the elderly (14), increased risk of infection (15, 16), and cardiovascular disease (17, 18). While some studies also associated T- cell senescence with a decreased risk for acute kidney rejection post-transplant (19–21), others have found differentiated T cells as biomarkers for acute rejection in a subgroup of patients (22) and long-term graft dysfuction in another group (23). Senescence is characterized by phenotypic changes including low CD4/CD8 ratio, reduced naïve and increased differentiated memory T cells (24), and the accumulation of terminally differentiated and highly cytotoxic CD28–/CD57+ T cells (25), as well as functional deficits including decreased responsiveness to stimulation and reduced proliferative capacity (9). Through chronic antigenic exposure, cytomegalovirus (CMV) appears to be a major driver of this process (26), however, age (8, 27) and chronic kidney disease (CKD) (28–30) have also been associated with similar changes, which are not reversed by kidney transplantation (31). An immune risk profile (IRP) has been proposed which has been associated with increased infectious complications post-transplant and mortality in the elderly (32–34). The IRP has been variably defined to include many of the above findings of immune senescence.

Natural killer (NK) cells represent early effector cells of the innate immune system and serve as the first line of defense against nascent neoplasms or viral infections. Similar to T cells, NK cells are affected by CMV, age (35) and the uremic state. Yet there is scarce evidence that age or CKD directly alters NK cell phenotype or compromises NK cell function. Healthy elderly present slightly enhanced proportions of NK cells, mostly driven by an increase in the more mature cytotoxic CD56^dim^ NK cell subset (36–41), while frequencies of the immature CD56^bright^ NK cell subset decrease with age (42). Functionally, diminished NK cell cytotoxicity has been reported in the elderly (39), and expression of the inhibitory receptor KLRG1 on mature CD56^dim^ NK cells increases with age (43). CD57 expression also serves as a marker of terminal differentiation in NK cells (44–46). Proportions of CD57+ NK cells are enhanced in the elderly. However, the direct effects of aging on NK cells are difficult to discriminate from those mediated by CMV infection, which significantly reshapes the NK cell repertoire. In particular, CMV replication promotes the expansion of NK cells expressing the activating NKG2C receptor (47–51). CD57 is frequently co-expressed on the NKG2C+ NK cell subset, and surface expression of both receptors has been associated with antigen-experienced and mature NK cells (51). Accordingly, CD57 and NKG2C are co-expressed on a significant portion of the memory-like NK cell subset lacking the FcR intracellular gamma signaling chain (FcγRΔg NK cells) and endowed with significantly enhanced antibody-dependent effector functions (52–54). Expression of other cell surface receptors on NK cells, such as CD27, has been proposed to mark functionally distinct subsets of NK cells, yet whether their expression reflects specific stages of human NK cell maturation remains unclear (55, 56). Furthermore, to date, little is known of NK cell contributions to IRP. Previous studies focusing on alterations of NK cells in patients with CKD report conflicting results, yet recent data strongly suggest a decrease in overall NK cell numbers in IRP+ (15) or dialyzed transplant candidates (57). However, phenotypic and functional characteristics of an optimal aged NK cell population associated with low viral reactivation and graft rejection remain unclear.

Lastly, determining IRP in a transplant candidate has the potential to better guide immune suppression after transplant. There are currently no clinical tests available to measure IRP. IRP has been previously determined based on a composite of serology and T cell phenotyping assays. We sought to determine if ImmuKnow®- an FDA approved clinical test for measuring overall T cell function in transplant recipients—can be used as a single clinical test that correlates closely to IRP to identify pre transplant patients at greater risk for infectious complications post-transplant.

## Hypothesis/Objective

While lymphocyte immune senescence has been described in ESRD, CKD, and post-transplant populations, it must be noted that patients listed for kidney transplant, especially elderly listed patients, represent a highly select population. We hypothesized that in addition to NK cells significantly contribute to immune senescence, in addition to T lymphocytes. We therefore sought to evaluate whether distinct NK cell signatures might be used in combination with T cell profiles, chronologic age, and other clinical information as markers of immune senescence, and to test whether T-cell based IRP definitions also correlate with distinct NK cell signatures.

## Materials and Methods

### Patients

This study was approved by Beth Israel Deaconess Medical Center Institutional Review Board. At our institution, 65 years older are the ages when kidney transplant candidates are considered as elderly candidates. In total, 59 subjects were recruited into five groups: (1) Healthy controls, (2) <65 years of age with CKD not on dialysis, (3) ≥65 years of age with CKD not on dialysis, (4) <65 years of age on dialysis, (5) ≥65 years of age on dialysis. Healthy controls were defined as individuals aged >18 years old with an estimated glomerular filtration rate (eGFR) >60 and no albuminuria. CKD patients were defined as adults listed for kidney transplant, but not yet requiring renal replacement therapy. Dialysis patients were defined as adults listed for kidney transplant and currently requiring renal replacement therapy. Patients with active infections, known systemic autoimmune conditions, those who have previously received immunosuppressant therapy, chemotherapy or a prior transplant were excluded. Patients were approached at their Transplant clinic visits and consented. Blood was collected from each subject. Healthy controls were approached at their Pre surgical visits for their elective surgery procedures. The IRP was defined as CMV seropositivity and CD4/CD8 ratio <1 (58) or being in the highest quintile of CD8+ senescent (CD28–/CD57+) T cells (14).

### Lymphocyte Phenotyping

A 100 μL aliquot of fresh whole blood was stained using a master mix of fluorescently-conjugated antibodies targeting the following human markers: CD3 (BD Biosciences SP34.2), CD20 (BD Biosciences L27), CD4 (BD Biosciences L200), CD8 (BD Biosciences RPA-T8), CD45RO (Beckman Coulter UHCL1), CD45RA (BD Biosciences 5H9), CD197/CCR7 (BD Biosciences 150503), CD28 (ThermoFisher 28.2), and CD57 (BD Biosciences NK-1). Following a 20 min/4°C staining incubation, automated red blood cell lysis was performed on the stained samples using a TQ-Prep™ Workstation (Beckman Coulter). Samples were then twice washed with 1X Dulbecco's Phosphate-Buffered Saline (Gibco), and subsequently fixed for cytometric analysis in 1.5% formaldehyde. Multicolor flow cytometry was then performed on the samples using a LSRII flow cytometer (BD Biosciences), and results were analyzed using FlowJo software (Treestar).

Specific cell populations were gated according to the following strategy: T-cells were identified using forward vs. side scatter, excluded of doublets, and further gated on CD3+ cells using a CD20 vs. CD3 plot to exclude B-cells. To gate CD3+/CD4+ and CD3+/CD8+ subpopulations most accurately, a Boolean gating strategy was designed directly off the CD3+ parent population. The effector cell subpopulation was defined for the CD3+/CD4+ and CD3+/CD8+ populations using Boolean gating for the CD3+/CD197+/CD45RA-/CD45RO+ cells. In a similar manner, the effector memory (EM), central memory (CM), and naive subpopulations of the CD3+/CD4+ and CD3+/CD8+ populations were defined using Boolean gating for the CD3+/197CD–/CD45RA–/CD45RO+, CD3+/CD197+/CD45RA–/CD45RO+, and CD3+/CD197+/CD45RA+/CD45RO– cells, respectively. Finally, senescent cells were defined for each group by applying Boolean gating for CD3+/28CD–/CD57+ cells to all subpopulations defined above.

To analyze NK cells, a 100 μL aliquot of fresh whole blood was stained using a master mix of fluorescently-conjugated antibodies. NK cells were defined as lymphocytes that were 3CD– (BioLegend UCHT1), 14CD– (BioLegend M5E2), and 19CD– (BioLegend HIB19). NK cell subpopulations were then identified based on their expression of CD56 (BioLegend HCD56) and/or CD16 (Pharmingen 3G8), namely bulk NK cells including CD56^dim^ (3CD–CD56+CD16+), CD56^bright^ (3CD–CD56+16CD–), and CD56neg (3CD−56CD–CD16+). Additional antibodies targeting the following human markers were used to assess their expression in NK cells: CD25 (eBioscience BC96), NKG2C (R&D Systems 134591), KLRG1 (BioLegend 2F1), CD2+ (Pharmingen RPA2.10), CD57 (BioLegend HCD57), CD27 (BioLegend 0323), FcεRIγ- (Millipore Sigma). Samples were fixed and analyzed using a LSRII flow cytometer (BD Biosciences), and data analyzed using FlowJo software 7.6.5 (Treestar).

### Flow Cytometric Analysis of NK Cell Function

To quantify NK cell function, thawed peripheral blood mononuclear cells (PBMC)s were incubated at 1 × 10^6^ cells/mL in RPMI-1640 supplemented with 10% fetal bovine serum, 2 mM L-glutamine, 100 mg/mL streptomycin, 100 U/mL penicillin, and 1 ng/mL rhIL-15 without stimulus or in the presence of 2 μg/mL CMV pp65 peptide pool (NIH AIDS reagents), 2 μg/mL BK virus capsid protein VP1 peptide pool (15 mer overlapping by 11, JPT), a combination of 50 ng/mL rhIL-12+100 ng/mL rhIL-18, HLA-I-deficient K562 or 721.221 cells at effector-to-target ratio of 10:1. To measure NK-cell mediated antibody-dependent cellular cytotoxicity (ADCC) function, p815 cells (a mouse leukemic cell line) were co-cultured with 1 mg/ml p815-specific antibody (Abcam) for 1 h and then washed twice. PBMCs were then stimulated with p815 cells or p815 cells plus p815-specific antibodies at an effector-to-target ratio of 10:1. PBMCs were stimulated for 6 h in the presence of 5 uL/mL CD107a-BV786 antibody (H4A3, BD Biosciences) and 1 μL/mL GolgiStop (monensin; BD Biosciences) prior to surface staining using the following anti-human monoclonal antibodies from BD Biosciences: CD3 (SP34-2), CD14 (M5E2), CD19 (HIB19), CD56 (B159), CD16 (3G8), fixed, permeabilized (BD Biosciences), and finally stained for intracellular interferon using IFN-γ-FITC (B27; BD Biosciences). Multiparameter flow cytometric analysis was performed on an LSRII instrument (BD Biosciences).

### ImmuKnow® Assay

The ImmuKnow® assay was performed by Viracor-Eurofin laboratory (Lee's Summit, MO) in accordance with published protocol (59). This assay determines total ATP released from CD4+ T cells with *ex vivo* mitogen stimulation by phytohemaglutinin-L. The assay has been approved by the United States Food and Drug Administration as a measurement total CD4+ T cell response in transplant recipients.

### Data Analysis

Data analysis was performed using STATA statistical software, version 14 (College Station, Texas). Continuous data was analyzed first utilizing the 5 original patient cohorts as predictors utilizing a Kruskal-Wallis test. Association between categorical variables was measured via Fisher's Exact test. In order to evaluate the contributions of each individual parameter on the outcome variables, univariable regression screen was performed. Any significant variables were placed into a multivariable linear regression. A partial least square discriminant analysis (PLSDA) using the mixomics R package (http://mixomics.org/methods/pls-da/) was performed to determine which features contribute to IRP. PLS-DA uses covariance to identify linear combinations of independent or latent variables that best differentiate between the different groups. Each variable is assigned a score, which can be visualized in the latent variable space (score plots). Latent variable loadings (loadings plots) can then be used to identify biomarker profiles associated with different groups. The prediction power of each set of variables was assessed using area under the curve (AUC) implemented in the mixomics package (60).

## Results

A portion of these data were presented at the American Transplant Congress in 2018 (61).

### Patients

Patient clinical characteristics are compared in Table 1. There were no significant differences between the study groups except for their age and the group with <65 and on dialysis had a large proportion of patients with diabetes as the cause of their CKD. We analyzed the CKD stages for the two groups not on dialysis. Within each group about half was in stage IV and half in stage V. There was no significant difference between the two groups. As expected the estimated glomeruli filtration rate (eGFR) were significantly lower in the two groups on dialysis.

**Table 1:** Patient demographics.

	**Healthy control**	**<65 CKD**	**≥65 CKD**	**<65 dialysis**	**≥65 dialysis**	***P***
*N*	12	12	11	12	12	
Age (years)	57.25	53.58	69.36	46.33	69.5	<0.01
Sex (*N*)						0.08
Female	8	4	2	5	2	
Male	4	8	9	7	10	
Race (*N*)						0.49
Caucasian	7	6	8	8	8	
Black/AA	4	3	1	3	4	
Hispanic	0	0	0	1	0	
Asian	1	3	2	0	0	
Dialysis Modality (*N*)						
HD				7	8	1.0
PD				5	4	
Dialysis Vintage (Months)				31.4	33.2	0.85
CKD Cause (*N*)						0.18
Diabetes		3	2	8	6	
HTN		2	4	2	2	
PKD/Congenital		2	3	2	0	
GN		2	0	0	1	
Other/Unknown		3	2	0	3	
eGFR (mL/min/1.73m^2^)		14	14	8.5	9	0.004
Diabetes (*N*)						0.22
No	8	9	8	4	6	
Yes	4	3	3	8	6	
CMV Serostatus (*N*)						0.91
Negative	4	6	4	6	5	
Positive	8	6	7	6	7	

*HD, Hemodialysis; PD, Peritoneal dialysis; HTN, Hypertension; PKD, polycystic kidney disease; GN, Glomerulonephritis*.

### Immune Risk Profile

**Figure 1:**
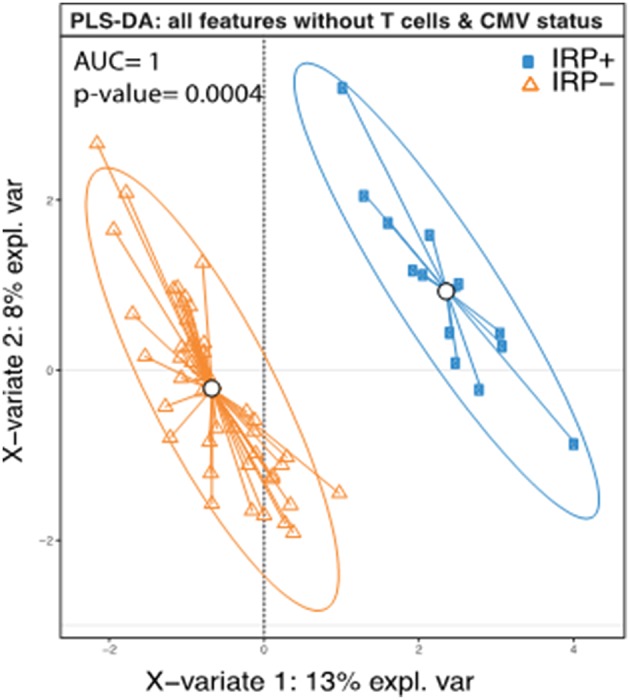
Complete separation of IRP+ and IRP– groups indicate contributions from both clinical and immune factors aside from those used to define IRP. PLS-DA plot of IRP+ and IRP– patients using the collected immune data except those that were used to determine IRP (CMV status, CD4 and CD8, and CD8+ senescent (28CD–/CD57+) cells. Each dot on the plot represents a subject, where blue represents IRP+ samples and orange represents IRP– samples. Confidence ellipses for each group were plotted to highlight the strength of the discrimination (confidence level set to 95%). Groups centroids were represented in the center of each group with circles.

Based on the definition of IRP, as CMV seropositivity and CD4/CD8 ratio <1 (58) or being in the highest quintile of CD8+ senescent (28CD–/CD57+) T cells (14), weidentified 14 individuals as having the IRP (IRP+). The 14 individuals were equally divided amongst the 5 original study groups (*P* = 1.0). Of the 40 CMV seropositive patients, 14 were found to be IRP+ (35%). Of the patients in the highest quintile of CD8+ senescent cells, 0 were found to be CMV negative, which is significantly different from the other quintiles (*P* = 0.003). Caucasians were less likely to be IRP+ than black/AA (0.36; 0.15–0.58) or Asian (0.42; 0.09–0.75). No other factors including CKD/dialysis status, dialysis modality or age were associated. Diabetes was included due to its near significance on the univariable logistic regression. It should be noted that whites were significantly less likely to be CMV positive with only 17/39 (44%) being CMV positive compared to 16/19 (84%) blacks/AA and 5/6 (83.3%) Asians (*P* = 0.003) consistent with previous population descriptions (62). Racial disparities in IRP status persisted when comparing only CMV+ Caucasians to black/AA (0.35; 0.02–0.68), but did not persist for Asian patients. In order to test if any of the other patients features except those that were used to determine IRP (CMV status, CD4 and CD8, and CD8+ senescent (28CD–/CD57+) cells, could predict the IRP status, we performed a PLS-DA analysis using the IRP status as the outcome and all factors except those that were used to determine IRP, as predictors. This model was able to predict IRP with an accuracy of 1 and a *p*-value = 0.0004 (Figure 1) suggesting in addition to CMV status, CD4 and CD8, and CD8+ senescent (28CD–/CD57+) cells, other factors contribute significantly in defining IRP.

### Analysis of T Cell Phenotypes

On multivariable analysis, age and IRP+ but not CMV+/IRP– status were found to affect frequency of CD4+. The percent of CD4+ T cells with a naïve phenotype decreased in both IRP+ and dialysis patients (Table 2A). Age did not affect the proportion of CD4+ naïve cells. The proportions of CD4+ CM cells remained unaffected by any of the variables. IRP+ status is strongly associated with an increased proportion of CD4+ EM cells. The proportion of CD4+ effector cells were not influenced by CMV status, but IRP is associated with an increase of these cells and CKD associated with a decrease. The proportion of CD4+ T cells which are 28CD–/CD57+ indicating senescence did not differ between the 5 cohorts (Table 2B). They were substantially and only increased in IRP+ patients and were not affected by other factors. CD4+ naïve and CM senescent cells are very rare and seen essentially only in IRP+ patients. CD4+ EM and effector senescent cells are more common and significantly increased in IRP+ and CMV+/IRP– patients, but are not affected by age, CKD or dialysis status.

**Table 2:** Multivariable analysis of factors associated with CD4+ T cell phenotypes (A) and senescence markers (B).

**A**										
	**Total CD4+**		**CD4+** **Naive**		**CD4+** **CM**		**CD4+EM**		**CD4+** **Effector**	
	**Coef**	***P***	**Coef**	***P***	**Coef**	***P***	**Coef**	***P***	**Coef**	***P***
CMV+/IRP– (vs. CMV–)	−3.89	0.25	−6.89	0.052	−2.84	0.55	11.20	0.003	0.43	0.70
IRP+ (vs. CMV–)	−15.55	<0.001	−14.52	0.001	1.42	0.79	19.84	<0.001	2.42	0.059
Age (per year)	0.41	<0.001	–.08	0.47	0.05	0.74	−0.05	0.68	−0.04	0.28
CKD (vs. HC)	1.62	0.68	−7.81	0.07	−0.67	0.91	2.56	0.56	−3.00	0.025
Dialysis (vs. HC)	7.21	0.07	−12.93	0.003	−0.58	0.92	7.47	0.08	−2.82	0.03
**B**										
	**Total CD4+** **Sen**		**CD4+** **Naïve Sen**		**CD4+** **CM Sen**		**CD4+** **EM Sen**		**CD4+** **Effector Sen**	
	**Coef**	***P***	**Coef**	***P***	**Coef**	***P***	**Coef**	***P***	**Coef**	***P***
CMV+/IRP– (vs. CMV–)	1.75	0.27	0.004	0.83	0.03	0.36	3.72	0.10	11.06	0.07
IRP+ (vs. CMV–)	8.11	<0.001	0.10	<0.001	0.14	<0.001	14.49	<0.001	35.67	<0.001
Age (per year)	0.07	0.18	0.00	0.56	0	0.78	0.10	0.14	0.11	0.53
CKD (vs. HC)	−3.36	0.08	−0.04	0.08	0.03	0.41	−4.10	0.13	−13.17	0.07
Dialysis (vs. HC)	−1.96	0.30	−0.05	0.04	0.01	0.46	−1.57	0.55	−10.25	0.15

On multivariable analysis, IRP+ but not CMV+/IRP– was associated with an increase in CD8+ T-cells while age was associated with a decline (Table 3A). CD8+ naïve cells were reduced in both the CMV+/IRP– and IRP+ groups and were unaffected by age, CKD and dialysis status. CD8+ CM cells were decreased in IRP+ patients. CD8+ EM were not affected by any variable. CD8+ TEMRA cells were expanded in both CMV+/IRP– and IRP+ groups. The proportion of CD8+ T cells which are 28CD–/57+ indicating senescence was increased only in the IRP+ group, and slightly decreased with age (Table 3B). The CMV–/IRP+ group was not different from the CMV– group. CD8+ senescent cells were unaffected by CKD or dialysis status. CD8+ naïve senescent cells were rare and only decreased in IRP+ patients. CD8+ CM naïve senescent cells are also rare and slightly increased with age. CD8+ EM and TEMRA cells were markedly increased in IRP+ patients and not in CMV+/IRP– patients and were not affected by the other variables.

**Table 3:** Multivariable analysis of factors associated with CD8+ T cell phenotypes (A) and senescence markers (B).

**A**										
	**Total CD8+**		**CD8+** **Naive**		**CD8+** **CM**		**CD8+EM**		**CD8+** **TEMRA**	
	**Coef**	***P***	**Coef**	***P***	**Coef**	***P***	**Coef**	***P***	**Coef**	***P***
CMV+/IRP– (vs. CMV–)	3.09	0.34	−11.97	0.01	−2.54	0.22	−3.71	0.42	14.15	0.005
IRP+ (vs. CMV–)	12.80	0.001	−17.97	0.001	−5.80	0.02	−0.38	0.94	23.72	<0.001
Age (per year)	−0.35	0.001	−0.18	0.21	0.07	0.31	0.13	0.36	−0.03	0.81
CKD (vs. HC)	−1.21	0.75	−7.35	0.19	1.74	0.49	2.43	0.66	−1.55	0.79
Dialysis (vs. HC)	−6.39	0.09	−6.15	0.26	2.20	0.37	2.70	0.62	−4.10	0.47
**B**										
	**Total CD8 Sen**		**CD8 Naïve Sen**		**CD8 CM Sen**		**CD8 EM Sen**		**CD8 TEMRA Sen**	
	**Coef**	***P***	**Coef**	***P***	**Coef**	***P***	**Coef**	***P***	**Coef**	***P***
CMV+/IRP– (vs. CMV–)	0.07	0.98	0.40	0.22	−0.09	0.79	−1.18	0.55	−0.82	0.86
IRP+ (vs. CMV–)	27.67	<0.001	0.85	0.03	0.28	0.48	17.33	<0.001	36.33	<0.001
Age (per year)	0.04	0.60	0.02	0.12	0.02	0.07	0.09	0.13	−0.25	0.08
CKD (vs. HC)	−3.37	0.25	0.26	0.51	0.22	0.59	−1.91	0.42	−6.39	0.26
Dialysis (vs. HC)	−3.72	0.20	−0.28	0.46	0.07	0.86	−2.39	0.31	−4.69	0.39

### Proportions, Phenotype, and Function of NK Cells

We next evaluated if enhanced proportions of NK cells with phenotypic features reflecting terminal differentiation are associated with CMV serostatus, age, kidney disease, and dialysis in our cohort of renal transplant candidates. To do so, we determined frequencies of NK cells expressing markers of NK cell activation (CD25), as well as those indicative of differentiation (CD57, KLRG1, NKG2C, CD2, and CD27) or adaptive features (FcεRIγ) (Tables 4A,B). Multivariable analysis demonstrates that age is associated with an increase in NK cells, which is not affected by IRP or CKD status. There were no differences in CD2, CD25, or CD57 expression. CMV+/IRP– and IRP+ status both tended toward an increase in CD27+ CD56^bright^ NK cells, though this did not reach significance. Memory-like FcγRΔg NK cells were significantly increased in CKD but not in dialysis patients, and were not significantly affected by IRP status or age. KLRG1+ NK cells were significantly reduced in IRP+ patients but were unaffected by the other factors. NKG2C+ NK cells were significantly increased in dialysis patients but not affected by the other factors. To examine if race affected NK cell phenotypes in our cohort, we performed analysis of data from the Caucasian subjects only and did not show additional significant difference.

**Table 4:** Multivariable analysis of factors associated with NK cell phenotypes.

**A**										
	**NK Cells**		**CD2**		**CD25**		**CD27**		**CD27 on CD56**^**bright**^	
	**Coef**	***P***	**Coef**	***P***	**Coef**	***P***	**Coef**	***P***	**Coef**	***P***
CMV+/IRP–	−2.29	0.19	−0.28	0.94	−9.84	0.22	−2.98	0.13	−2.65	0.06
IRP+	−0.96	0.64	4.21	0.32	−6.77	0.39	−5.41	0.02	−2.92	0.08
Age (per year)	0.13	0.02	0.10	0.37	0.18	0.44	−0.05	0.40	−0.05	0.27
CKD (vs. HC)	0.02	0.99	−3.09	0.48	−1.89	0.81	1.68	0.47	0.66	0.69
Dialysis (vs. HC)	−3.26	0.12	1.0	0.81	−8.32	0.33	1.90	0.40	0.89	0.57
**B**										
	**CD57**		**FC**γ**RIneg**		**KLRG1**		**NKG2C**			
	**Coef**	***P***	**Coef**	***P***	**Coef**	***P***	**Coef**	***P***		
CMV+/IRP–	4.37	0.28	−6.47	0.56	−4.39	0.22	9.94	0.20		
IRP+	4.73	0.31	−15.94	0.22	−10.01	0.01	1.98	0.82		
Age (per year)	0.10	0.40	−0.43	0.23	0.04	0.74	−0.02	0.93		
CKD (vs. HC)	8.65	0.07	27.37	<0.05	−2.68	0.53	4.16	0.65		
Dialysis (vs. HC)	7.12	0.13	3.51	0.79	−0.32	0.94	23.11	0.01		

**Figure 2:**
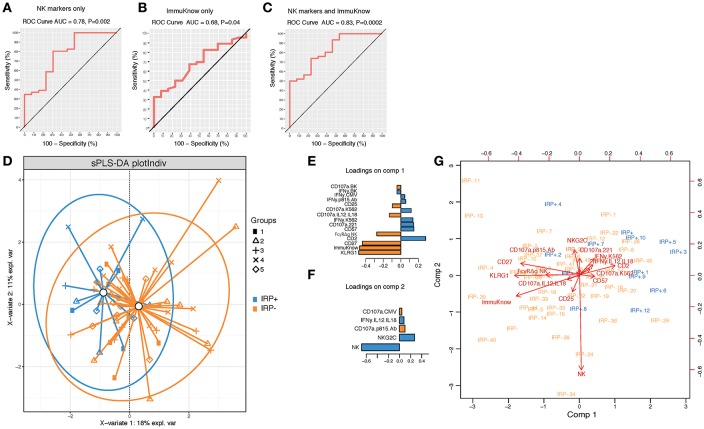
Multivariate analysis using partial least-square discriminant analysis (PLS-DA) shows NK activation markers and ImmuKnow® variable segregate IRP– and IRP+ subjects. **(A–C)** Receiver operating characteristic (ROC) curves for NK markers alone **(A)**, ImmuKnow® only (and NK markers + ImmuKnow® **(C)**. Receiver operating characteristic (ROC) curve of PLS-DA model predicting IRP outcome. Receiver operating characteristic (ROC) curve showing the accuracy of the PLS-DA model in predicting IRP+ vs. IRP– outcomes using NK markers alone **(A)**, ImmuKnow® alone **(B)**, or NK markers with ImmuKnow® combined **(C)**. The true positive rate (sensitivity) is plotted in function of the false positive rate (100-specificity). The area under the ROC curve (AUC) measures how well the model distinguishes IRP+ from IRP– subjects for each analysis. **(D)** Individual PLS-DA plot of IRP+ and IRP– patients using NK markers and ImmuKnow® as predictors and IRP status as outcome. Each dot on the plot represents a subject, where blue represents IRP+ samples and orange represents IRP– samples. Confidence ellipses for each group are plotted to highlight the strength of the discrimination (confidence level set to 95%). Groups centroids were represented in green circles. **(E,F)** Loading weights of each feature selected on the first the second component of the multivariate model. Loading plots represent the top features selected on the first **(E)** and the second **(F)** component of the PLS-DA model using tune sPLS-DA with color indicating the class with a maximal mean expression value for each feature. **(G)** Features correlation circle plots. Correlation circle plots display the correlation between measured features and latent components. Each variable coordinate is defined as the Pearson correlation between the original data and latent components 1 and 2. The contribution of each feature to each component is represented by features proximity to the large circle of radius 1. This plot shows also the correlation between features (clusters of features). The cosine angle between any two points represent the correlation (negative, positive, or null) between two features. A threshold of 0.5 is set to remove weaker correlations and to plot only features with major importance. Strongly correlated variables are projected in the same direction from the origin. The distance from the origin is correlated to the strength of the association.

To further determine if NK cell profiles can discriminate IRP+ and IRP– patients and can be used in combination with ImmuKnow® values as a surrogate predictor of IRP, we performed a PLSDA analysis using NK cell phenotypic and functional data alone and in combination with ImmuKnow® values. Our multivariate analysis revealed that NK cell features, including proportions of bulk NK cells, and percentages of NK cells expressing KLRG1, CD27, CD57, and NKG2C, predicted IRP with an accuracy of 0.78 and a *p*-value of 0.002 (Figure 2A). When using ImmuKnow® alone we achieved an accuracy of 0.68 and *p*-value of 0.04 (Figure 2B). Interestingly, by combining the ImmuKnow® variable and NK cell features, we enhanced the prediction power of our model by reaching an accuracy of 0.83 and a *p*-value of 0.0002 (Figure 2C). This analysis showed that combining NK features and ImmuKnow® segregated IRP+ and IRP– groups where 18% of the variability between these two groups was explained by the first component and 11% by the second component of the PLSDA (Figure 2D). Several NK features were significant on both components and associated with either IRP+ or IRP– (Figures 2E,F).

Overall, this analysis showed that NK cell signatures contributed significantly to the IRP status where KLRG1, FcγRΔg NK cells, and CD27 were associated with the IRP– status and CD2, CD57, NKG2C, and proportions of NK cells were associated with the IRP+ status (Figure 2G). Furthermore, NK cell function against BK or CMV peptide pools also contributed to the NK cell-driven differentiation of IRP, with the IRP+ status being associated with IFN-γ production and the IRP– status with degranulation, as measured by CD107a upregulation, in response to those viral antigens (see Supplementary Material).

### T Cell Function Measured by ImmuKnow® Assay

The ImmuKnow® assay was not significantly different across the 5 study groups (*P* = 0.08). In order to evaluate the role of CMV positivity as well as the IRP, we performed further analysis comparing CMV+ patients who did not display the IRP (CMV+/IRP–) and IRP+ to CMV– patients (Table 5). A multivariable analysis demonstrated that increased age (−4.70; *P* = 0.005) was significantly associated with decreased ImmuKnow® value (Table 5). Dialysis patients tended to have a higher ImmuKnow® value (111.40; *p* = 0.08), and CMV seropositivity was associated with lower ImmuKnow® value (−74.21, *p* = 0.16), though these did not reach significance. Other demographic and clinical factors, including CKD not requiring dialysis, were not associated with ImmuKnow® values.

**Table 5:** Multivariable analysis showed ImmuKnow® results were significantly associated with age and IRP.

	**ImmuKnow®**	
	**Coef**	***P***
CMV+/IRP– (vs. CMV–)	−74.21	0.16
IRP+ (vs. CMV–)	−154.73	0.01
Age (per year)	−4.70	0.005
CKD (vs. HC)	54.70	0.39
Dialysis (vs. HC)	111.40	0.08

## Discussion

In this study, we sought to determine if NK cells contribute to immune senescence in renal transplant candidates. We have demonstrated that CMV serostatus, age, and dialysis all contribute toward phenotypic compositions of the T cell compartment. We have also shown that not all patients who are CMV seropositive display an IRP. These patients are in some ways phenotypically similar to CMV negative patients and in others display an intermediate phenotype between CMV– and IRP+ patients. Thus, while CMV latency seems necessary to produce the IRP, it is not sufficient.

While CMV drives senescence, the IRP is concentrated in only a few individuals with the most exaggerated T-cell phenotypic changes. The proportion of T cells which are CD4+ is markedly decreased and the proportion which are CD8+ is increased, leading to a decreased CD4/CD8 ratio. Naïve and CM cells in both CD4 and CD8 lineages are reduced while there is an increase in more differentiated EM and TEMRA cells, consistent with prior descriptions of IRP (24). CD4+/28CD–/CD57+ senescent T cells, which are associated with unstable atherosclerotic plaques (63, 64) and clinical coronary atherosclerosis (17, 18, 65–67) are seen essentially only in IRP+ patients.

CD8+ senescent T cells, which are associated with a reduced likelihood of allograft rejection (20, 21) are markedly increased in IRP patients, as it is part of the definition of IRP, but more importantly, are not increased in CMV+/IRP– patients when compared to CMV– patients. This suggests that recipient CMV seropositivity in of itself is not an adequate marker of T cell senescence. Other contributing factors to CD8+ T cell senescence could include exposure to other latent viral infections such as herpes simplex viruses and human polyomaviruses, and immune modulation by T regulatory cells (68).

In this study, we included a panel of antibodies to specifically characterize NK cell subpopulations in kidney transplant candidates. As previously reported, we found decreased proportions of NK cells in our cohort of patients, although the difference with healthy controls did not reach statistical significance. Unexpectedly, we found that proportions of NK cells expressing markers previously associated with CMV latency were not increased in CMV–seropositive patients compared to seronegative subjects, suggesting that in this cohort of renal transplant candidates, other factors than CMV might significantly influence the differentiation status of NK cells. For instance, dialysis was associated with a marked increase in NKG2C positive NK cells, and further investigations are warranted to determine if this is a direct effect of dialysis, and if dialysis can modulate the ability of NK cells to control viral infections. Similarly, CKD but not CMV seropositivity was associated with augmented proportions of the memory-like FcγRΔg NK cell subset, suggesting that other viral infections or clinical factors might drive the generation of this NK cell subset. Importantly, the IRP+ status could be discriminated based on NK cell expression of the terminal maturation marker CD57 and of the adaptive markers NKG2C and CD2 (69–72). This observation suggests that in patients currently defined as positive for IRP, NK cells are characterized by a terminally differentiated and CMV–driven phenotype.

We examined the contributions of NK cells to immune senescence, and, to our knowledge, provide the first report of distinct NK cell signatures associated with IRP status, dialysis or kidney disease in kidney transplant candidates. Whether the NK cell features identified in this study can predict the IRP status and are associated with reduced NK cell-mediated control of viral infections will need to be confirmed in additional cohorts. These observations highlight the importance of considering additional factors than those currently used to determine the overall immune status of those patients. A thorough evaluation of immune senescence will need to include additional facets of the immune system, such as B cells and antibodies (73), macrophages, and activation and exhaustion markers of the immune cells. Future studies compiling these additional immune features are needed to build IRP and guide precise personalized transplant immune suppression.

We had hypothesized that natural age is not predictive of immune senescence and that immunological age would be a better predictor. Our data showed that age is associated with a relative expansion of the CD4+ T cell subset and decrease in the CD8+ T cell subset, but did not seem to significantly alter CD3+ T cell subsets nor senescence markers. It was, however, associated with a decrease in ImmuKnow® value. Thus, in this highly select population, chronological age does not predict T cell senescence, though is associated with reduced T cell function as measured via the ImmuKnow® assay.

Dialysis was associated with a substantial decline in CD4+ naïve T cells which seems to be exacerbated by the IRP. However, CKD and dialysis status does not seem to significantly alter the CD8+ T cell pool nor significantly affect T cell senescence. Dialysis status was also associated with a non-significant increase in ImmuKnow® value, which is at odds with the known decrease in proliferative capacity in such patients, but may instead reflect the aberrant state of T cell activation (74).

We evaluated the potential of using ImmuKnow® as a clinical tool to identify subjects with IRP+, who may be at increased risk for acquiring infection post-transplant. ImmuKnow® value was significantly reduced in IRP+ patients, but not in CMV+/IRP– patients, consistent with the reduced T cell function seen in immune senescence. This may indicate that CMV infection affects CD8+ T cells more than CD4+ T cells. ImmuKnow® measures total CD4+ T cell function by quantifying ATP release, which does not take into account CD8+ T cells, including the TEMRA cells associated with immune senescence. In elderly kidney transplant candidates, ImmuKnow® could potentially be used to identify those who are IRP+, as demonstrated by our data. However, the wide variability in ImmuKnow values may mean that a larger cohort is needed for further determination.

There are several limitations in this study. We prospectively consented and enrolled 65 subjects, the number of subjects in each group was small. Given the heterogeneous nature of human subject studies, a larger sample size may be needed to better account for these differences. We had consented subjects consecutively in pre kidney transplant clinics and in the outpatient elective surgery testing center without gender preference. This resulted in an imbalance of more female in the healthy control group as compared to the male dominance in the other groups. NK cell activity and thymic function differs between the two genders (75, 76). This could have attributed bias to our results.

## Conclusion

Immune senescence is associated with increased risk of infection and decreased risk of rejection post kidney transplant. CMV seems to drive T cell senescence in some, but not all patients who have latent infection. The subset of patients with exaggerated T cell phenotypic changes of senescence called the IRP are not easily identified by age, CKD or dialysis status or other clinical and demographic factors. The ImmuKnow® assay can be used to identify kidney transplant candidates with IRP. NK cell features are affected by CKD and dialysis status more so than age or CMV latency and may help explain the susceptibility of such patients to infections and malignancies. NK cells also contribute to immune senescence, and NK cell signatures combined with ImmuKnow® assay have the potential to predict IRP in the study population.

Traditional risk factors for a senescent or aged immune system correlate well with actual phenotypic changes of senescence associated with increased risk of infection, cardiovascular disease, death and lower risks of allograft rejection. In order to assess senescence, T cell phenotyping appears to be necessary but not sufficient. Phenotype and function of additional components of the immune system such as NK cells, potentially in combination with the Immuknow® assay, could be used to accurately define immune senescence.

## Data Availability

The raw data supporting the conclusions of this manuscript will be made available by the authors, without undue reservation, to any qualified researcher.

## Author Contributions

DD, MA, KM, SK, SJ, and CT contributed conception and design of the study. DD, KM, JG, EG, CH, and SM enrolled subjects into the study, collected primary data, and performed the immunology experiments. MA performed the statistical analysis. DD wrote the first draft of the manuscript. MA, KM, JG, EG, CH, SM, SK, SJ, and CT wrote sections of the manuscript. All authors contributed to manuscript revision and read and approved the submitted version.

### Conflict of Interest Statement

The authors declare that the research was conducted in the absence of any commercial or financial relationships that could be construed as a potential conflict of interest.
