# Supplementary figures and images for: NK Cells Contribute to the Immune Risk Profile in Kidney Transplant Candidates
Source: Front Immunol. 2019 Aug 23;10:1890. doi: 10.3389/fimmu.2019.01890 (PMC6716214; doi:10.3389/fimmu.2019.01890)

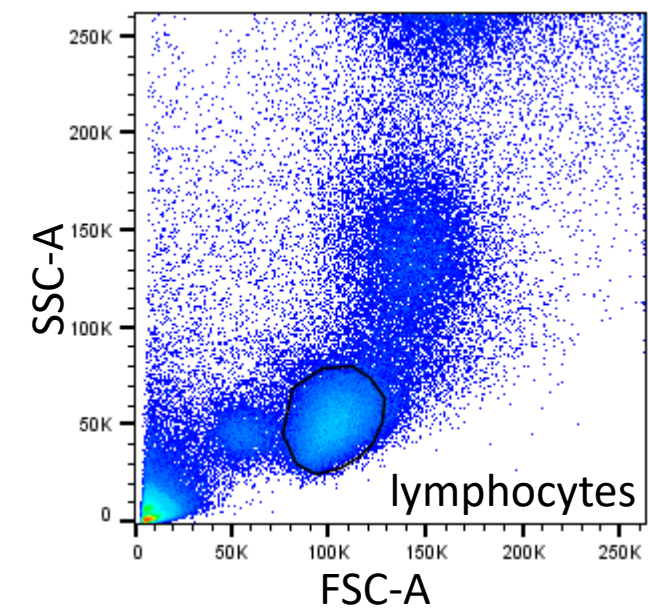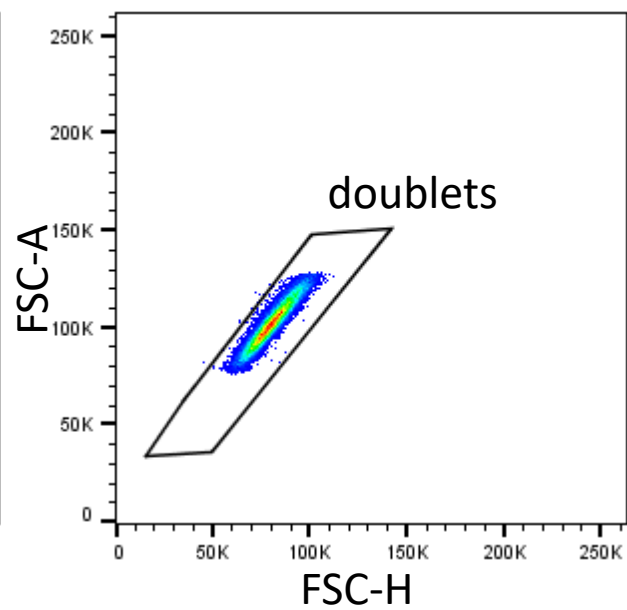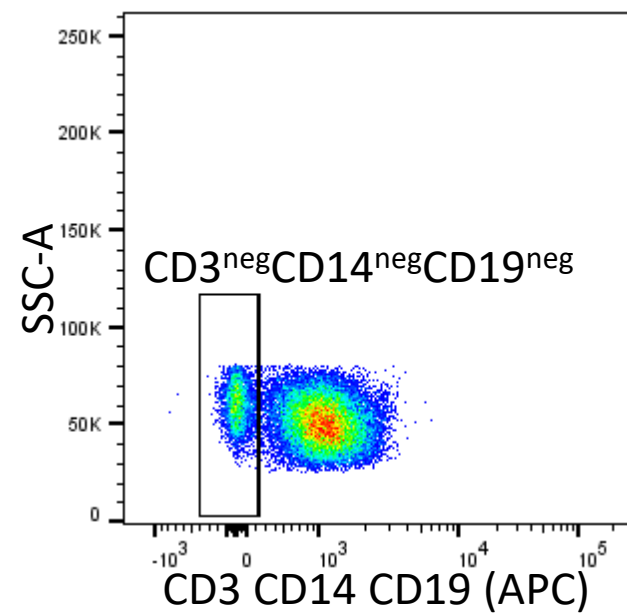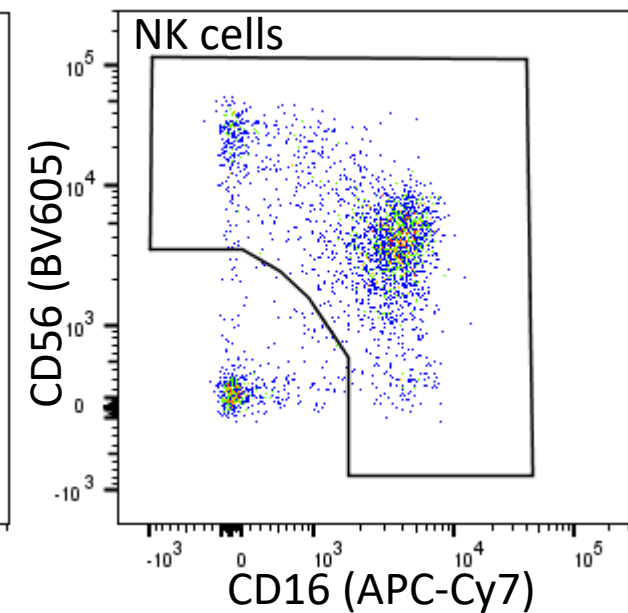

Supplement: Supplementary file 1 [file Data_Sheet_1.PDF]

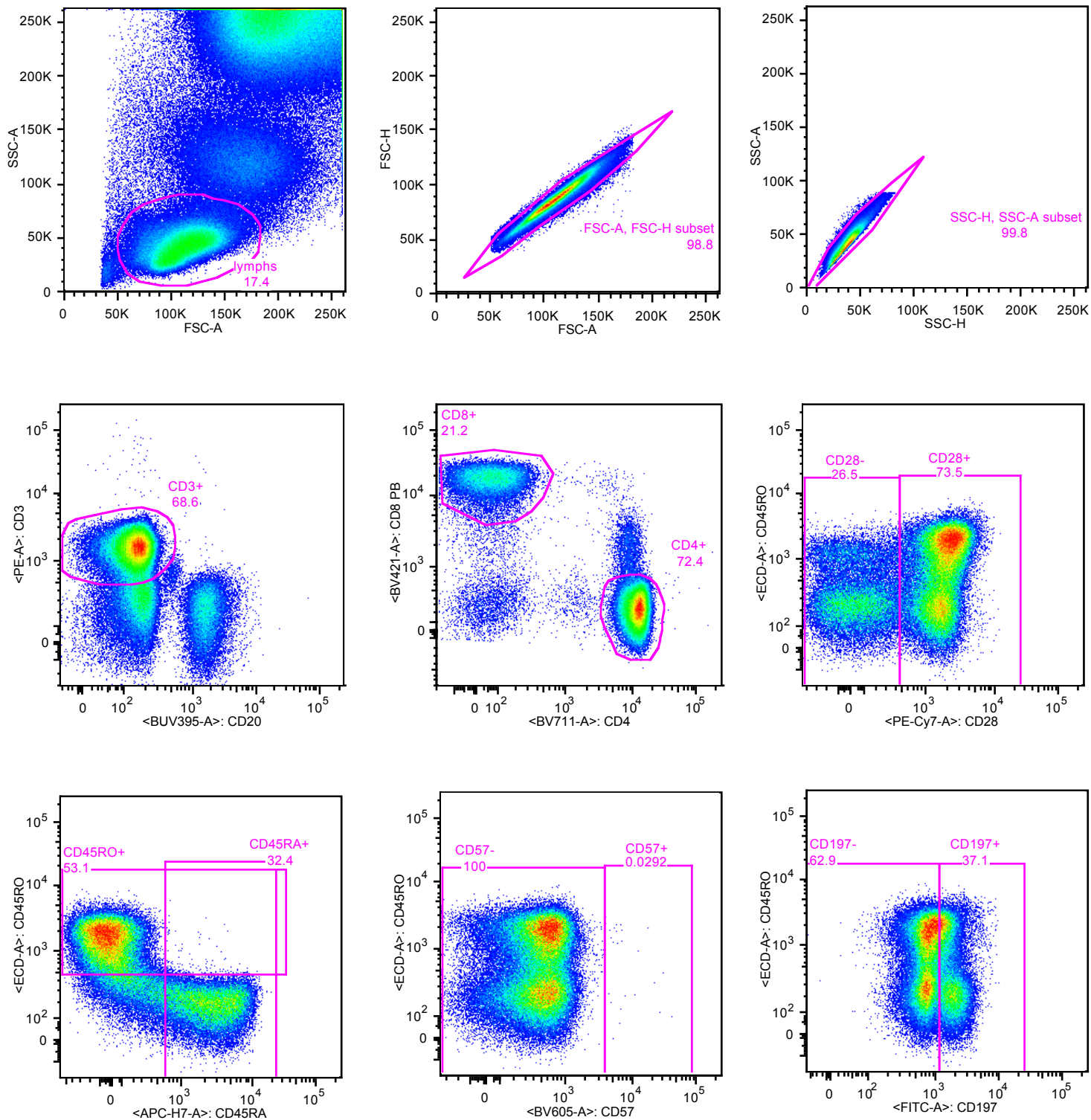

Supplement: Supplementary file 2 [file Data_Sheet_2.PDF]
