# Supplementary material for: NK Cells Contribute to the Immune Risk Profile in Kidney Transplant Candidates
Source: Front Immunol. 2019 Aug 23;10:1890. doi: 10.3389/fimmu.2019.01890 (PMC6716214; doi:10.3389/fimmu.2019.01890)
Supplement: Supplementary file 3 [file Data_Sheet_3.docx]

**Supplementary figure 3.**

1. **NK cell phenotype in IRP- and IRP+ subjects**

1. **NK cell functions as measured by IFN-γ production (b.1) and CD107a upregulation (b.2) in response to indicated stimuli in IRP- and IRP+ subjects.**

**b.1**

**b.2**
